# Supplementary material for: Inflammation in VTA Caused by HFD Induces Activation of Dopaminergic Neurons Accompanied by Binge-like Eating
Source: Nutrients. 2022 Sep 16;14(18):3835. doi: 10.3390/nu14183835 (PMC9502544; doi:10.3390/nu14183835)

Supplementary Materials

Table S1. List of qRT-PCR primers used in this study

Common abbreviation	Forward primer (5′→ 3′)	Reverse primer (5′→ 3′)
TNF-α	CATCTTCTCAAAATTCGAGTGACA A	TGGGAGTAGACAAGGTACAACCC
IL1B	CGACAAAATACCTGTGGCCT	TTCTTTGGGTATTGCTTGGG
PTP1B	GCGCTTCTCCTACCTGGCTGTCAT	ACGTGCTCGGGTGGAAGGTCTA
Socs3	GTGAAGAGGCAGTAGCA	TCTCCTAGCCCCACATAG
IL10	GGTTGCCAAGCCTTATCGGA	ACCTGCTCCACTGCCTTGCT
Iba1	TGATCCCAAATACAGCAATGA TGAG	TCCAGCATTCGCTTCA AGGAC
CD11b	CGGAAAGTAGTGAGAGAACTGTTTC	CTTATAATCCAAGGGATCACCGAATTT
Emr1	AATCGCTGCTGGTTCAATACAG	CCAGGCAAGGAGGACAGAGTT
CD68	CTTCCCACAAGCAGCACAG	AATGATGAGAGGCAGCAAGAGA
GFAP	AACGACTATCGCCGCCAACTG	CTCTTCCTGTTCGCGCATTTG
GLAST	CTGTTTCGGAATGCCTTCGTT	TCACCTCCCGGTAGCTCATTT
cFos	CTGTCAACACACAGGACTTTT	AGGAGATAGCTGCTCTACTTTG
FosB	AGGCAGAGCTGGAGTCGGAGAT	GCCGAGGACTTGAACTTCACTCG
DAT	TGGCTGTTGGTGTAAAGTGG	CCAAAAGACGGCAATATGGT
TH	TTGAAGGAACGGACTGGCTT	GAAACACACGGAAGGCCAGA
D1R	GTAGCCATTATGATCGTCAC	GATCACAGACAGTGTCTTCAG
D2R	GCAGCCGAGCTTTCAGAGCC	GGGATGTTGCAGTCACAGTG
GAPDH	AGGTCGGTGTGAACGGATTTG	TGTAGACCATGTAGTTGAGGTCA

Table S2. The details of statistics used in this study.

Figure	Panel	Number of samples	Test used	F/t/p value and degrees of freedom (df)	Post hoc test	Significance
2A	Body Weight	WT = 14, KO = 12	Two-way ANOVA assessed by repeated measures	Time: F (13, 312) = 725, $p < 0.05$<br>Genotype: F (1, 24) = 0.2696, ns<br>Interaction: F (13, 312) = 0.0505, ns	Sidak's multiple comparison	WT vs KO, ns
2B	Body Weight	WT = 12, KO = 6	Two-way ANOVA assessed by repeated measures	Time: F (1.423, 22.77) = 177.2, $p < 0.05$<br>Genotype: F (1, 16) = 0.05773, ns<br>Interaction: F (13, 208) = 0.1827, ns	Sidak's multiple comparison	WT vs KO, ns
2C	Food intake	WT = 13, KO = 14	Unpaired t-test	t = -1.129, df = 25		WT vs KO, ns
2D	Food intake	WT = 7, KO = 6	Unpaired t-test	t = -0.789, df = 11		WT vs KO, ns
2E	Feed efficiency	WT = 13, KO = 14	Unpaired t-test	t = -0.489, df = 25		WT vs KO, ns
2F	Feed efficiency	WT = 7, KO = 6	Unpaired t-test	t = 0.510, df = 11		WT vs KO, ns
2G	Blood Glucose	WT = 7, KO = 11	Unpaired t-test	t = 0.105, df = 16		WT vs KO, ns
2H	Blood Glucose	WT = 11, KO = 5	Unpaired t-test	t = 0.861, df = 14		WT vs KO, ns
2I	White adipose tissue	WT = 7, KO = 10	Unpaired t-test	t = 0.927, df = 15		WT vs KO, ns
2J	White adipose tissue	WT = 11, KO = 5	Unpaired t-test	t = 0.191, df = 14		WT vs KO, ns
4A	Food intake	WT = 6, KO = 8	Two-way ANOVA assessed by repeated measures	Time: F (1.446, 17.35) = 89.76, $p < 0.05$<br>Genotype: F (1, 12) = 0.2250, ns<br>Interaction: F (2, 24) = 1.284, ns	Sidak's multiple comparison	WT vs KO, ns
4B	Food intake	WT = 13, KO = 11	Two-way ANOVA assessed by repeated measures	Time: F (1.636, 36) = 128.4, $p < 0.05$<br>Genotype: F (1, 22) = 14.34, $p < 0.05$<br>Interaction: F (2, 44) =0.2759, ns	Sidak's multiple comparison	WT vs KO (30 min), $p < 0.05$<br>WT vs KO (60 min), $p < 0.05$<br>WT vs KO (120 min), $p < 0.05$
4C	Food intake	WT = 8, KO = 9	Two-way ANOVA assessed by repeated measures	Time: F (1.475, 22.13) = 18.75, $p < 0.05$<br>Genotype: F (1, 15) = 0.09574, ns<br>Interaction: F (2, 30) =0.2240, ns	Sidak's multiple comparison	WT vs KO, ns
4D	Food intake	WT = 12, KO = 10	Two-way ANOVA assessed by repeated measures	Time: F (1.165,23.30) = 80.04, $p < 0.05$<br>Genotype: F (1, 20) = 6.163, $p < 0.05$<br>Interaction: F (2, 40) = 7.820, $p < 0.06$	Sidak's multiple comparison	WT vs KO (30 min), ns<br>WT vs KO (60 min), $p < 0.05$<br>WT vs KO (120 min), $p < 0.05$

4E	pAKT/AKT	CD WT = 7, CD KO = 6<br>HFD WT =6, HFD KO = 6	Two-way factorial ANOVA	Group: F (1, 21) = 0.3454, p < 0.05<br>Genotype: F (1, 21) = 41.14, ns<br>Interaction: F (1, 21) = 0.9275, ns	Sidak's multiple comparison	WT-CD vs WT-HFD, p < 0.05<br>HFD-WT vs HFD-KO, ns
4F	pAKT/AKT	CD WT = 5, CD KO = 6<br>HFD WT =6, HFD KO = 6	Two-way factorial ANOVA	Group: F (1, 5) = 17.43, p < 0.05<br>Genotype: F (1, 5) = 2.349, ns<br>Interaction: F (1, 4) = 9.003, p< 0.05	Sidak's multiple comparison	WT-CD vs WT-HFD, p < 0.05<br>HFD-WT vs HFD-KO, p < 0.05
5A	TNF-α mRNA	WT = 8, KO = 7	Unpaired t-test	t = 2.060, df = 13		WT vs KO, ns
	IL1B mRNA	WT = 8, KO = 8	Unpaired t-test	t = 1.306, df = 14		WT vs KO, ns
	PTP1B mRNA	WT = 8, KO = 7	Unpaired t-test	t = -0.019, df = 13		WT vs KO, ns
	socs3 mRNA	WT = 8, KO = 8	Unpaired t-test	t = 0.183, df = 14		WT vs KO, ns
	IL10 mRNA	WT = 7, KO = 8	Unpaired t-test	t = 0.307, df = 13		WT vs KO, ns
5B	TNF-α mRNA	WT = 7, KO = 6	Unpaired t-test	t = 2.491, df = 10		WT vs KO, p < 0.05
	IL1B mRNA	WT = 7, KO = 7	Unpaired t-test	t = -0.491, df = 12		WT vs KO, ns
	PTP1B mRNA	WT = 7, KO = 7	Unpaired t-test	t = 4.749, df = 12		WT vs KO, p < 0.05
	socs3 mRNA	WT = 7, KO = 7	Unpaired t-test	t = 1.555, df = 6.770		WT vs KO, ns
	IL10 mRNA	WT = 7, KO = 6	Unpaired t-test	t = -2.361, df = 11		WT vs KO, p < 0.05
5C	Iba1 mRNA	WT = 8, KO = 7	Unpaired t-test	t = 2.360, df = 13		WT vs KO, p < 0.05
	CD11b mRNA	WT = 8, KO = 8	Unpaired t-test	t = 1.146, df = 14		WT vs KO, ns
	Emr1 mRNA	WT = 8, KO = 8	Unpaired t-test	t = -0.294, df = 14		WT vs KO, ns
	CD68 mRNA	WT = 8, KO = 8	Unpaired t-test	t = 0.287, df = 14		WT vs KO, ns
	GFAP mRNA	WT = 8, KO = 8	Unpaired t-test	t = 1.180, df = 14		WT vs KO, ns
	GLAST mRNA	WT = 8, KO = 8	Unpaired t-test	t = 0.497, df = 14		WT vs KO, ns
5D	Iba1 mRNA	WT = 6, KO = 6	Unpaired t-test	t = 2.507, df = 10		WT vs KO, p < 0.05
	CD11b mRNA	WT = 7, KO = 7	Unpaired t-test	t = 2.743, df = 12		WT vs KO, p < 0.05
	Emr1 mRNA	WT = 7, KO = 7	Unpaired t-test	t = 0.769, df = 12		WT vs KO, ns
	CD68 mRNA	WT = 7, KO = 7	Unpaired t-test	t = -1.189, df = 12		WT vs KO, ns
	GFAP mRNA	WT = 7, KO = 7	Unpaired t-test	t = 2.640, df = 12		WT vs KO, p < 0.05
	GLAST mRNA	WT = 7, KO = 7	Unpaired t-test	t = 1.620, df = 7.168		WT vs KO, ns
6A	DAT mRNA	WT = 7, KO = 8	Unpaired t-test	t = 2.434, df = 13		WT vs KO, p < 0.05
	cFos mRNA	WT = 7, KO = 6	Unpaired t-test	t = 1.116, df = 11		WT vs KO, ns
	ΔFosB mRNA	WT = 7, KO = 7	Unpaired t-test	t = -0.189, df = 12		WT vs KO, ns
	TH mRNA	WT = 8, KO = 8	Unpaired t-test	t = -0.092, df = 14		WT vs KO, ns
6B	DAT mRNA	WT = 7, KO = 7	Unpaired t-test	t = 5.788, df = 12		WT vs KO, p < 0.05

	cFos mRNA	WT = 7, KO = 7	Unpaired t-test	t = 1.116, df = 12		WT vs KO, ns
	ΔFosB mRNA	WT = 8, KO = 6	Unpaired t-test	t = 0.905, df = 9.238		WT vs KO, ns
	TH mRNA	WT = 7, KO = 6	Unpaired t-test	t = 1.321, df = 11		WT vs KO, ns
6C	DAT mRNA	WT = 6, KO = 5	Unpaired t-test	t = 0.8782, df = 9		WT vs KO, ns
	cFos mRNA	WT = 7, KO = 7	Unpaired t-test	t = 1.705, df = 12		WT vs KO, ns
	ΔFosB mRNA	WT = 7, KO = 7	Unpaired t-test	t = -0.215, df = 12		WT vs KO, ns
	D1R mRNA	WT = 7, KO = 7	Unpaired t-test	t = -2.065, df = 12		WT vs KO, ns
	D2R mRNA	WT = 7, KO = 7	Unpaired t-test	t = -1.016, df = 12		WT vs KO, ns
6D	DAT mRNA	WT = 6, KO = 7	Unpaired t-test	t = 0.2764, df = 11		WT vs KO, ns
	cFos mRNA	WT = 8, KO = 8	Unpaired t-test	t = -0.932, df = 14		WT vs KO, ns
	ΔFosB mRNA	WT = 8, KO = 8	Unpaired t-test	t = -0.499, df = 14		WT vs KO, ns
	D1R mRNA	WT = 7, KO = 8	Unpaired t-test	t = 0.410, df = 13		WT vs KO, ns
	D2R mRNA	WT = 7, KO = 8	Unpaired t-test	t = -0.211, df = 13		WT vs KO, ns
6E	DAT mRNA	WT = 7, KO = 6	Unpaired t-test	t = 1.486, df = 11		WT vs KO, ns
	cFos mRNA	WT = 7, KO = 6	Unpaired t-test	t = 2.530, df = 11		WT vs KO, $p < 0.05$
	ΔFosB mRNA	WT = 7, KO = 7	Unpaired t-test	t = 0.165, df = 12		WT vs KO, ns
	D1R mRNA	WT = 7, KO = 6	Unpaired t-test	t = -1.685, df = 11		WT vs KO, ns
	D2R mRNA	WT = 7, KO = 7	Unpaired t-test	t = -0.211, df = 12		WT vs KO, ns
6F	DAT mRNA	WT = 8, KO = 7	Unpaired t-test	t = 2.398, df = 12		WT vs KO, $p < 0.05$
	cFos mRNA	WT = 7, KO = 7	Unpaired t-test	t = 2.599, df = 12		WT vs KO, $p < 0.05$
	ΔFosB mRNA	WT = 8, KO = 7	Unpaired t-test	t = 2.186, df = 13		WT vs KO, $p < 0.05$
	D1R mRNA	WT = 8, KO = 7	Unpaired t-test	t = 2.163, df = 13		WT vs KO, $p < 0.05$
	D2R mRNA	WT = 8, KO = 7	Unpaired t-test	t = 2.304, df = 13		WT vs KO, $p < 0.05$

Figure S1. Visual appearance of the stomachs of mice after binge-like eating

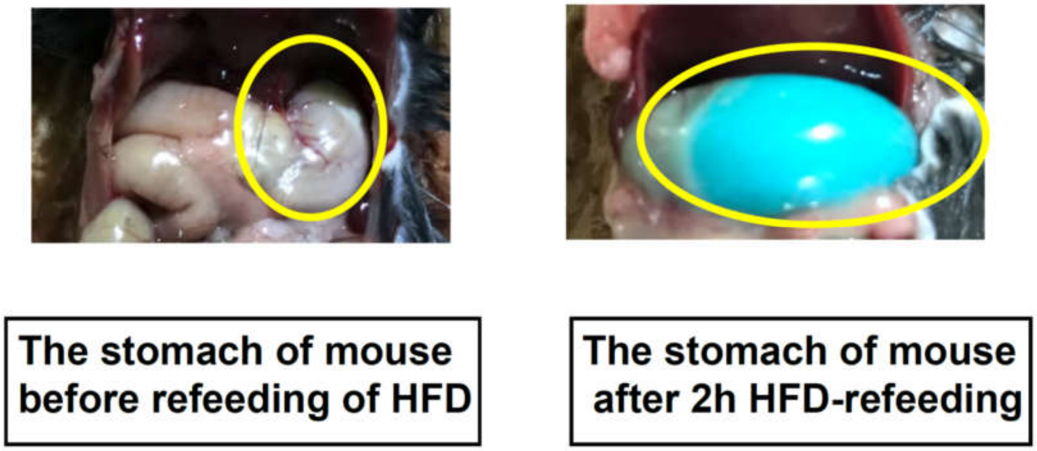

Figure S2. Uncropped images of Western Blots

Figure 4E

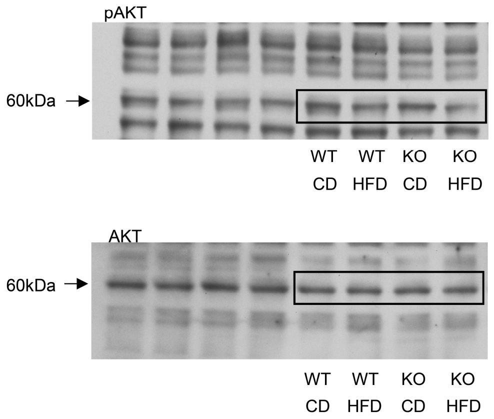

Figure 4F

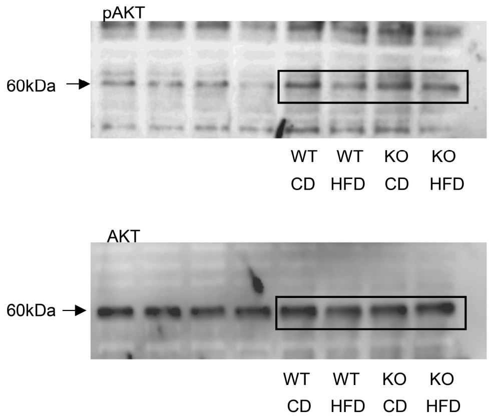

Supplement: Supplementary file 1 [file nutrients-14-03835-s001.zip › nutrients-1912613-supplementary.pdf]
